# Cucurbitacin B inhibits the stemness and metastatic abilities of NSCLC via downregulation of canonical Wnt/β-catenin signaling axis

**DOI:** 10.1038/srep21860

**Published:** 2016-02-24

**Authors:** Samriddhi Shukla, Sonam Sinha, Sajid Khan, Sudhir Kumar, Kavita Singh, Kalyan Mitra, Rakesh Maurya, Syed Musthapa Meeran

**Affiliations:** 1 Laboratory of Cancer Epigenetics, Division of Endocrinology, CSIR-Central Drug Research Institute, Lucknow, India; 2 Division of Medicinal and Process Chemistry, CSIR-Central Drug Research Institute, Lucknow, India; 3 Electron Microscopy Unit, SAIF, CSIR-Central Drug Research Institute, Lucknow, India; 4 Academy of Scientific and Innovative Research (AcSIR), New Delhi, India

## Abstract

Lack of effective anti-metastatic drugs creates a major hurdle for metastatic lung cancer therapy. For successful lung cancer treatment, there is a strong need of newer therapeutics with metastasis-inhibitory potential. In the present study, we determined the anti-metastatic and anti-angiogenic potential of a natural plant triterpenoid, Cucurbitacin B (CuB) against non-small cell lung cancer (NSCLC) both *in vitro* and *in vivo*. CuB demonstrated a strong anti-migratory and anti-invasive ability against metastatic NSCLC at nanomolar concentrations. CuB also showed significant tumor angiogenesis-inhibitory effects as evidenced by the inhibition of migratory, invasive and tube-forming capacities of human umbilical vein endothelial cells. CuB-mediated inhibition of angiogenesis was validated by the inhibition of pre-existing vasculature in chick embryo chorio-allantoic membrane and matrigel plugs. Similarly, CuB inhibited the migratory behavior of TGF-β1-induced experimental EMT model. The CuB-mediated inhibition of metastasis and angiogenesis was attributable to the downregulation of Wnt/β-catenin signaling axis, validated by siRNA-knockdown of *Wnt3* and *Wnt3a*. The CuB-mediated downregulation of Wnt/β-catenin signaling was also validated using 4-(methylnitrosamino)-1-(3-pyridyl)-1-butanone (NNK)-induced lung tumorigenesis model *in vivo*. Collectively, our findings suggest that CuB inhibited the metastatic abilities of NSCLC through the inhibition of Wnt/β-catenin signaling axis.

Metastasis contributes to the malignant progression of cancer, thereby adding to the fatality of the disease. The delayed diagnosis of lung cancers facilitates metastasis of primary lung tumors to secondary metastatic sites such as bones and brain. Tumor angiogenesis also plays a very important role in the progression as well as metastatic spread of solid tumors including lung tumors. Therefore, for effective lung cancer treatment, efficient anti-metastatic and anti-angiogenic agents are required. Natural compounds have been shown to have enormous potential as anti-proliferative as well as anti-metastatic agents against multiple cancer types^1^,^2^,^3^,^4^,^5^. We have recently shown the strong anticancer potential of Cucurbitacin B (CuB) through epigenetic modulation against non-small cell lung cancer (NSCLC) *in vitro* as well as *in vivo*^6^. Recent reports have also shown that CuB effectively inhibits migratory and invasive potential of breast cancer and liver cancer cells^7^,^8^. However, the effect of CuB on lung cancer metastasis as well as tumor angiogenesis has yet not been explored.

Canonical Wnt/β-catenin signaling pathway is well known to be functionally important in the regulation of cellular proliferation, differentiation, motility as well as polarity. Aberrations in this pathway are implicated in the pathogenesis of multiple tumor types, including lung cancer^9^,^10^,^11^. Aberrant promoter methylation has been linked to the upregulation of Wnt/β-catenin pathway in lung cancer^10^. Wnt binding to the frizzled (FZD) receptors guides nuclear translocation of β-catenin, further leading to the activation of genes involved in the cellular proliferation, metastasis as well as angiogenesis. Wnt/β-catenin signaling pathway, together with the Notch and Hedgehog pathways, has been shown to be involved in self-renewal of hematopoietic as well as cancer stem cells^10^.

In the present study, we sought to determine the anti-migratory and anti-invasive potential of CuB on metastatic human NSCLC cells and in the 4-(methylnitrosamino)-1-(3-pyridyl)-1-butanone (NNK)-induced lung tumorigenesis mice model. We also assessed the effect of CuB treatment on the angiogenic potential of human umbilical vein endothelial cells (HUVECs) *in vitro*. For the assessment of anti-angiogenic properties of CuB *ex vivo* and *in vivo,* we utilized chick embryo chorio-allantoic membrane (CAM) and matrigel plug assays, respectively. CuB inhibited the β-catenin expression as well as nuclear localization in the NSCLC cells as well as NNK-induced lung tissues. CuB-mediated downregulation of β-catenin signaling led to the downregulation of in the protein expression of β-catenin target genes. Role of Wnt/β-catenin pathway in CuB-mediated anti-metastatic effects was validated by siRNA knockdown of *Wnt3/3A*. Our findings suggest that CuB inhibits migratory and invasive abilities of both moderately as well as highly metastatic NSCLC cells through the downregulation of Wnt/β-catenin signaling axis. In addition, CuB exhibits potent anti-angiogenic activity *in vitro*, *ex vivo* as well as *in vivo*.

## Results

### CuB inhibits the migratory potential of human NSCLC cells

**Figure 1:**
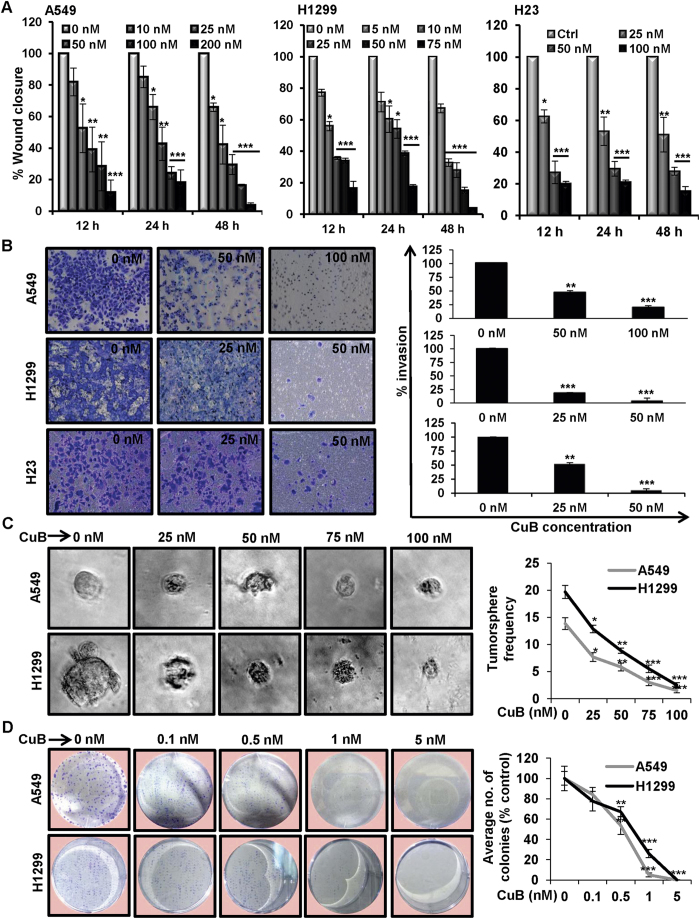
CuB suppresses cellular migration, invasion and stemness in NSCLC cells at sub-IC_50_ concentrations. (**A**) Wounds were created in subconfluent A549 (Panel **A**), H1299 (Panel **B**) and H23 (Panel **C**) cells and cells were treated with indicated concentrations of CuB for 12, 24 and 48 h. Data were expressed as percent wound closure in treatment groups compared to control. (**B**) A549, H1299 and H23 cells were seeded in matrigel-coated chambers and treated with indicated concentrations of CuB for 48 h. Data were expressed as percent invasion in treatment groups compared to control (**C**) A549 and H1299 cells were seeded in ultra-low attachment 96-well plates in 10 replicates with variable concentrations of CuB. The numbers of tumorspheres were counted and represented as average number of tumorspheres per well. (**D**) The average number of CuB-treated A549 and H1299 colonies containing ≥50 cells were counted and represented as percent of control. Each treatment was repeated in triplicates. Results were obtained from three independent experiments, mean ± SEM. *p < 0.05, **p < 0.01, ***p < 0.001 versus control.

To assess the effects of CuB treatment on the migratory potential of different human NSCLC cells, we performed wound healing assays in moderately metastatic A549 as well as highly metastatic H1299 and H23 lung cancer cells with different concentrations of CuB. We found that CuB induced a significant dose- and time-dependent inhibition of cellular migration in A549 cells, starting with the concentrations of 25 nM at 12 and 24 h, and 10 nM at 48 h, respectively. Similarly for highly metastatic H1299 and H23 cells, the significant dose- and time-dependent inhibition of cellular migration was observed starting with concentrations as low as 10 nM and 25 nM, respectively (p < 0.05) (Fig. 1A; Supplementary Fig. S1). These concentrations of CuB were much lesser than the IC_50_ concentrations for cellular proliferation and apoptosis against NSCLC, as reported by us, previously^6^. Therefore, these results indicate that CuB inhibits the migratory capability of NSCLC cells at sub-toxic concentrations.

### CuB inhibits the invasive capability and stemness of human NSCLC cells

We next analyzed the effects of CuB on the invading ability of the NSCLC cells. Moderately invasive A549 as well as highly invasive H1299 and H23 cells were treated with varying concentrations of CuB for 48 h. As shown in Fig. 1B, CuB exhibits significant dose-dependent inhibition of invasion in A549 at 50 nM and 100 nM concentrations (p < 0.01 and p < 0.001, respectively). In case of H1299 cells, invasion was significantly inhibited at CuB concentrations ≥25 nM (p < 0.001). Similarly, in H23 cells, significant dose-dependent inhibition of invasion was observed at concentrations starting with 25 nM of CuB. We performed tumorsphere assays in A549 and H1299 cells in serum-free, non-adherent conditions to assess the effects of CuB on the stemness of these cells. CuB significantly reduced the size and frequency of tumorspheres in both A549 and H1299 cells, in a dose-dependent manner (Fig. 1C). The effect of CuB on the *in vitro* colony forming potential of NSCLC cells was determined using anchorage-dependent colony formation assay. CuB significantly inhibited the colony formation in A549 and H1299 cells starting with a concentration of 0.5 nM, and at ≥5 nM CuB concentrations, no colonies were detected (Fig. 1D). Collectively, these results suggest that CuB dose-dependently inhibits the invasive ability as well as stemness of NSCLC cells.

### CuB inhibits endothelial cell migration, invasion and angiogenesis

**Figure 2:**
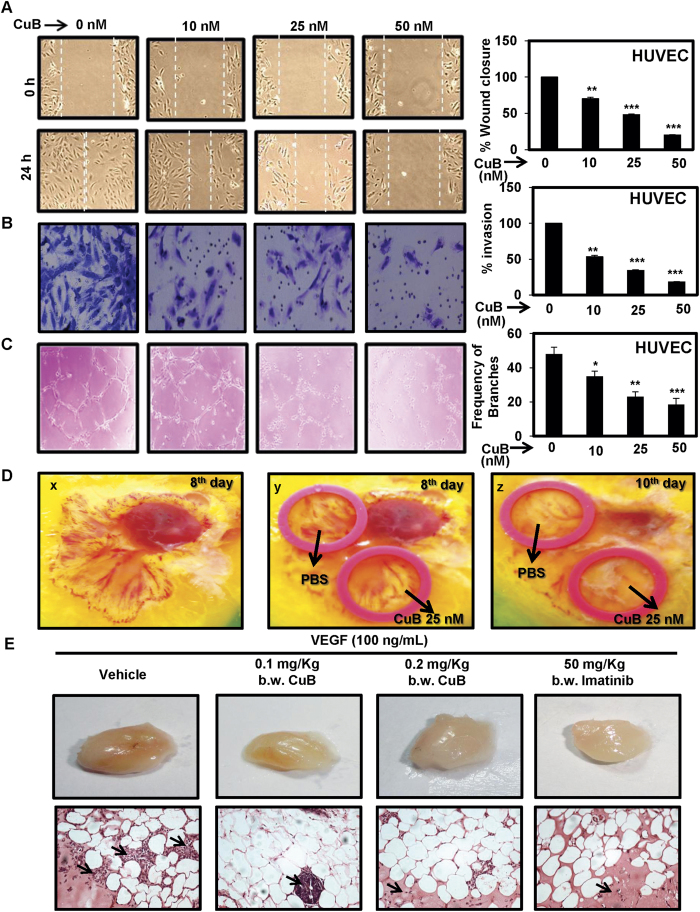
CuB inhibits angiogenesis *in vitro* and *ex vivo*. (**A**) Wound healing assays were performed in the subconfluent HUVECs. Images of wound closure were captured at 0 h and 24 h after treatment. Data were expressed as percent wound closure compared to control. (**B**) HUVECs were seeded onto the upper surface of the matrigel-coated chambers with indicated concentrations of CuB for 24 h. Images of invaded cells were captured and total numbers of invaded cells were counted in 10 different microscopic fields. Each treatment was given in triplicates and data were plotted as percent invasion compared to control. (**C**) HUVECs were seeded in matrigel-precoated 96-well plates and treated with indicated concentrations of CuB for 6 h. Images of tube formation from each group was captured and total number of nodes and branches were calculated. Each treatment was given in triplicates and results were represented as average number of nodes and branches present in each treatment group. (**D**) The anti-angiogenic potential of CuB was determined by performing chorio-allantoic membrane (CAM) assay. Images x & y show the CAM membrane exposed after 8 days incubation of fertilized egg. Image z shows the effect of 25 nM CuB on the pre-existing vasculature after two days of exposure. The images are representative of three independent experiments. (**E**) The *in vivo* anti-angiogenic potential of CuB was determined by implanting matrigel plugs in the right flank of BALB/c mice, and then by treating the mice with 0.1 mg/kg and 0.2 mg/kg b.w. doses of CuB. Imatinib at 60 mg/kg b.w. dose was used as positive control. Matrigel plugs from different animal groups were excised and photographed. H& E staining was performed to visualize the vascular formation. The arrows in the histopathological sections indicate the presence of endothelial cells. The images are representative of each group. In each case, results were obtained from three independent experiments, mean ± SEM. *p < 0.05, **p < 0.01, ***p < 0.001 versus control.

Endothelial cell migration is an essential step in tumor angiogenesis. To determine the effect of CuB on the endothelial cell migration, we preformed wound healing assay in HUVECs treated with varying concentrations of CuB. As shown in Fig. 2A,B, CuB at concentrations ≥10 nM significantly inhibited the migration and invasion of HUVECs at 24 h. Tubulogenesis is the exclusive ability of endothelial cells to form tube-like structures, which facilitate the formation of new blood vessels. We assessed the effect of CuB on the tube-formation ability of HUVECs; CuB dose-dependently inhibited the tube formation in HUVECs after 6–8 h, at concentrations ≥10 nM (p < 0.05; Fig. 2C). We also assessed the effect of CuB on *ex vivo* angiogenesis through CAM assay. CuB was shown to considerably inhibit the pre-existing vasculature after 2 days (Fig. 2D). We analyzed the effects of CuB on the inhibition of tumor angiogenesis in matrigel plugs *in vivo*. We found that 100 ng/mL VEGF induced the formation of blood vessels in the matrigel plugs. The intraperitonial administration of 0.1 and 0.2 mg/Kg b.w. CuB resulted in a dose-dependent inhibition of tumor angiogenesis in the VEGF-induced matrigel plugs. The standard angiogenesis inhibitor, imatinib also considerably inhibited the microvessel formation in these VEGF-induced matrigel plugs. Further, histological analysis of matrigel plugs confirmed the inhibition of functional vasculature followed by the CuB as well as imatinib treatment (Fig. 2E). Together, these results indicate that in addition to its anti-migratory and anti-invasive capabilities, CuB also exhibits strong anti-angiogenic potential, which might contribute to the anti-cancer properties of CuB in NSCLC.

### CuB inhibits β-catenin expression as well as nuclear localization in human NSCLC cells

**Figure 3:**
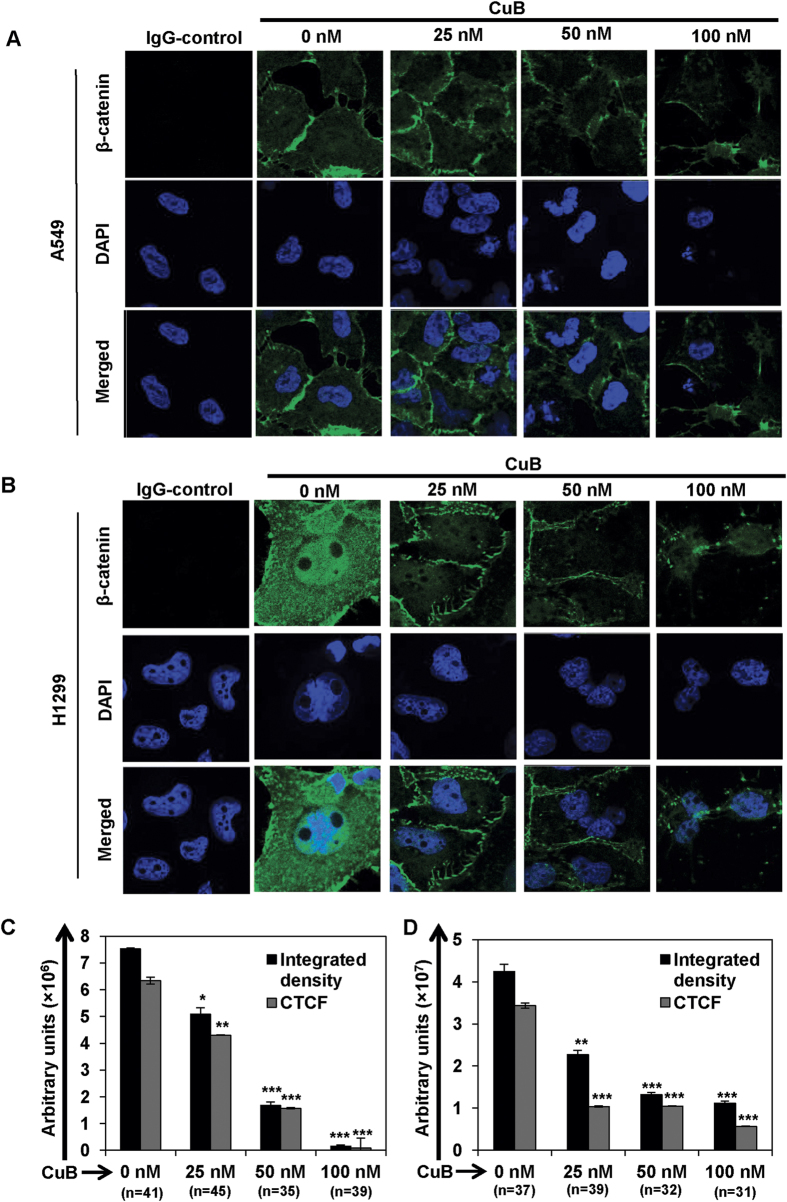
CuB inhibits the expression and nuclear translocation of β-catenin in NSCLC cells. A549 (Panel **A**) and H1299 (Panel **B**) cells were seeded on glass cover slips for 24 h and then treated with varying concentrations of CuB for 24 h. Endogenous cytoplasmic and nuclear β-catenin (FITC-green) localization was visualized by immunofluorescence followed by confocal imaging. DAPI was used as nuclear stain (blue). IgG control was used as the negative control. Images are representative of three-independent experiments. The graphs (Panels **C**,**D**) represent the raw integrated density as well as CTCF values in A549 and H1299 cells, respectively. The numbers in parenthesis are indicative of number of cells analyzed for each group.

Wnt/β-catenin signaling is essential for the lung tumorigenesis as well as maintenance of the stemness-characteristics in multiple cancer subtypes including lung cancer^9^,^12^,^13^. To ascertain the role of β-catenin behind the anti-metastatic and anti-stemness activity of CuB, we examined β-catenin expression as well as nuclear translocation through confocal immunofluorescence imaging in NSCLC cells. As shown in Fig. 3A,C, untreated A549 cells showed a higher β-catenin expression with lower nuclear localization. We observed a significant dose-dependent decrease in the cellular expression of β-catenin in A549 cells followed by CuB treatment. Moreover, the remaining β-catenin was found to be accumulated towards the cytoplasmic membrane. In case of H1299 cells, the untreated control cells displayed intense β-catenin localization both in the cytoplasm and nucleus compared to untreated A549 cells, which deciphers the role of β-catenin in the metastatic ability of these NSCLC cells. CuB treatment induced significant downregulation of the cytoplasmic expression and nuclear localization of β-catenin in a dose-dependent manner (p < 0.01; Fig. 3B,D). Similarly, we also found a significant dose-dependent decrease in the expression as well as nuclear localization of β-catenin in H23 cells followed by CuB treatment (p < 0.001; Supplementary Fig. S2A,C). These results suggest that downregulation of β-catenin expression as well nuclear localization are associated with the anti-metastatic and anti-angiogenic functions of CuB.

### CuB inhibits canonical β-catenin signaling in human NSCLC cells

**Figure 4:**
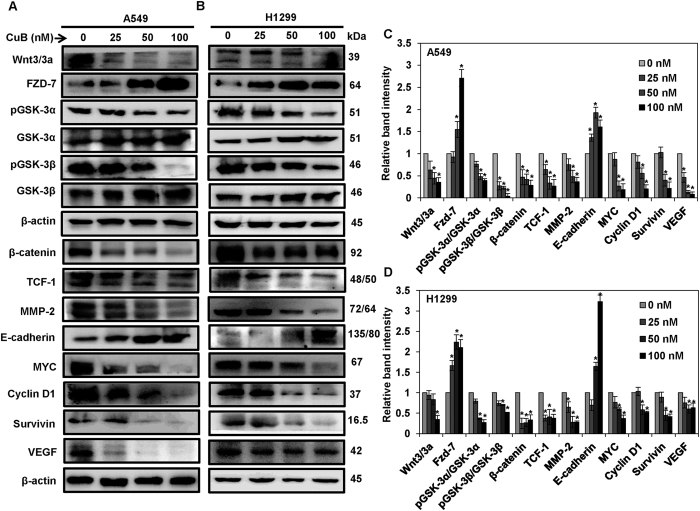
CuB inhibits the Wnt/β-catenin signaling in the NSCLC cells. A549 (Panel **A**) and H1299 cells (Panel **B**) were treated with 0–100 nM of CuB for 24 h and whole cell lysates were prepared. Western blot analysis was performed to analyze the expression of Wnt/β-catenin pathway proteins. Blots are representative of three independent experiments. Graphs in (Panel **C**,**D**) represent relative band intensities of proteins in A549 and H1299 cells normalized to β-actin. The results were obtained from three independent experiments, mean ± SEM. *p < 0.05 versus Control.

The nuclear translocation of β-catenin results in the activation of canonical Wnt/β-catenin signaling, leading to the transcriptional activation of downstream targets. Therefore, we analyzed the effect of CuB on the expression patterns of proteins involved in this pathway and the downstream targets of β-catenin. As shown in Fig. 4A–D, the expression of both Wnt3 and Wnt3a was found to be significantly inhibited by the CuB treatment, in both A549 and H1299 cells. Surprisingly, the expression of seven-pass transmembrane FZD-7 receptor was found to be significantly increased by the CuB treatment in both the cell lines. GSK-3, a key component of the Wnt/β-catenin signaling, is an important member of β-catenin degradation complex^14^. In mammalian cells, GSK-3 protein exists as two homologs, GSK-3α and GSK-3β. It has been established that both of these GSK-3 homologs function identically in the regulation of Wnt signaling^15^. GSK-3-mediated phosphorylation of β-catenin leads to its ubiquitination and subsequent proteasomal degradation. Phosphorylation of GSK-3α at serine 21 and phosphorylation of GSK-3β at serine 9 leads to inhibition of these proteins, leading to the activation of Wnt/β-catenin signaling^14^,^16^,^17^,^18^,^19^. We found loss of inhibitory phosphorylations at both GSK-3α and GSK-3β as well as upregulation of GSK-3α and GSK-3β expression in both A549 and H1299 cells. These events cumulatively result in an increased active GSK-3 activity, which culminates into β-catenin degradation. The expression of transcription factor TCF-1 was found to be downregulated in both A549 and H1299 cells. Matrix metallopeptidases (MMPs) are enzymes, which facilitate the cancer cell invasion and migration through the degradation of the extracellular matrix. MMP-2 is one such gelatinase, which is also directly regulated through the β-catenin expression. We found that CuB significantly inhibited the expression of MMP-2 in both A549 and H1299 cells. E-cadherin/β-catenin adhesion complex is critical for cell-cell adhesion and maintenance of normal and malignant tissue architecture. Loss of E-cadherin and β-catenin expression has been correlated with invasive phenotype in NSCLC^20^. We analyzed the effect of CuB treatment on the expression of E-cadherin and significant upregulation of E-cadherin expression was observed in case of both NSCLC cell lines. We also assessed the effect of CuB treatment on the cellular expression of other β-catenin target proteins such as MYC and Cyclin D1 in NSCLC cells. The expressions of pluripotency gene MYC and cellular proliferation marker Cyclin D1 were significantly downregulated with CuB treatment in both A549 and H1299 cells. Overexpression of β-catenin has been reported to induce VEGF expression, thereby augmenting tumor angiogenesis^21^,^22^. Survivin is an angiogenesis-related protein, downstream to β-catenin, which promotes VEGF-induced tumor angiogenesis^23^. CuB treatment led to significant downregulation of survivin expression in NSCLC cells. In accordance, CuB also inhibited VEGF expression significantly in both A549 and H1299 cells (p < 0.05; Fig. 4C,D). Collectively, these results suggest that the downregulation of Wnt/β-catenin signaling axis bestows the anti-metastatic and anti-angiogenic behavior on CuB against NSCLC.

### CuB inhibits TGF-β1-induced migration in A549 cells

**Figure 5:**
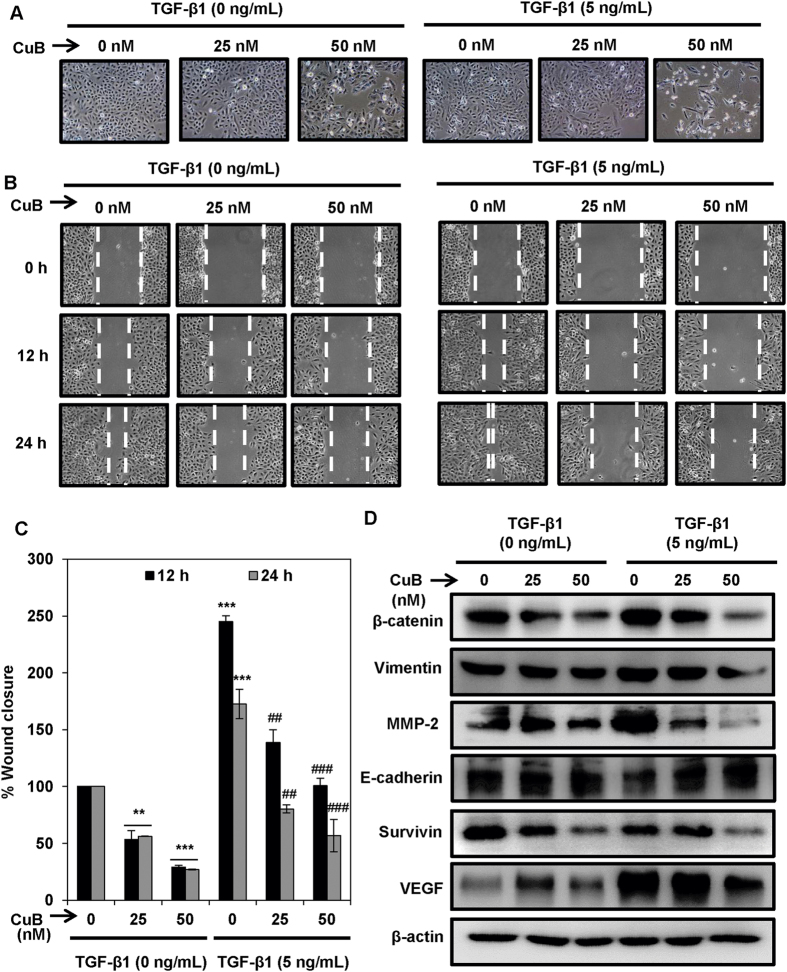
CuB treatment abrogates TGβ-1-induced EMT in A549 cells. (**A**) A549 cells were treated with TGF-β1 (5 ng/mL) for 24 h. TGF-β1 treated and untreated A549 cells were treated with 25 and 50 nM concentrations of CuB for 24 h. Morphological changes are shown in light microscopic images (100× magnification). (**B**,**C**) Wounds were created in the sub-confluent cultures of A549 cells and then cells were treated with TGF-β1 (5 ng/mL) and 25 and 50 nM CuB. Photomicrographs were captured at 0, 12 and 24 h after treatment and wound area was measured. Data were expressed as percent wound closure in the treatment groups compared to control. The results were obtained from three independent experiments, mean ± SEM. **p < 0.01 and ***p < 0.001 compared to untreated control, ^**##**^p < 0.01 and ^**###**^p < 0.001 compared to respective TGF-β1-treated control group. (**D**) A549 cells were treated as in (Panel **A**), and whole cell lysates were prepared. Western blot analysis was performed to analyze the expression of EMT markers. Blots are representative of three independent experiments.

We used TGF-β1 induced experimental epithelial-to-mesenchymal transition (EMT) model to analyze higher efficiency of CuB treatment in inhibiting the NSCLC cells with highly aggressive malignant behavior. The exposure to TGF-β1 induces EMT in moderately metastatic NSCLC A549 cells rendering mesenchymal phenotype in otherwise epithelial cells^24^,^25^. Control A549 cells exhibited characteristic epithelial phenotype with close cell-cell contacts, whereas TGF-β1-treated A549 cells displayed mesenchymal morphology, as characterized by their spindle-shaped appearance with large inter-cellular spaces and loss of cell-cell contacts. CuB marginally restored the epithelial morphology in TGF-β1-induced A549 cells by reducing the intercellular spaces and causing cell shrinkage (Fig. 5A). Next, we performed wound healing assay in TGF-β1-treated A549 cells. As shown in Fig. 5B,C, A549 cells treated with TGF-β1 showed aggravated migratory potential than the untreated A549 cells due to their mesenchymal phenotype. Further, CuB has significantly inhibited the TGF-β1-induced A549 migration in a dose-dependent as well as time-dependent manner at 25 nM and 50 nM concentrations compared with TGF-β1-untreated A549 cells of epithelial morphology. EMT is associated with increased expression of mesenchymal markers and decreased expression of epithelial markers. TGF-β1 induced the expression of mesenchymal markers such as β-catenin, MMP-2 and vimentin, while downregulated the expression of epithelial marker, E-cadherin. However, CuB treatment led to the downregulation of the mesenchymal markers and increased expression of E-cadherin in this experimental EMT-model (Fig. 5D).

### CuB inhibits metastasis and angiogenesis in NSCLC cells via Wnt/β-catenin signaling

**Figure 6:**
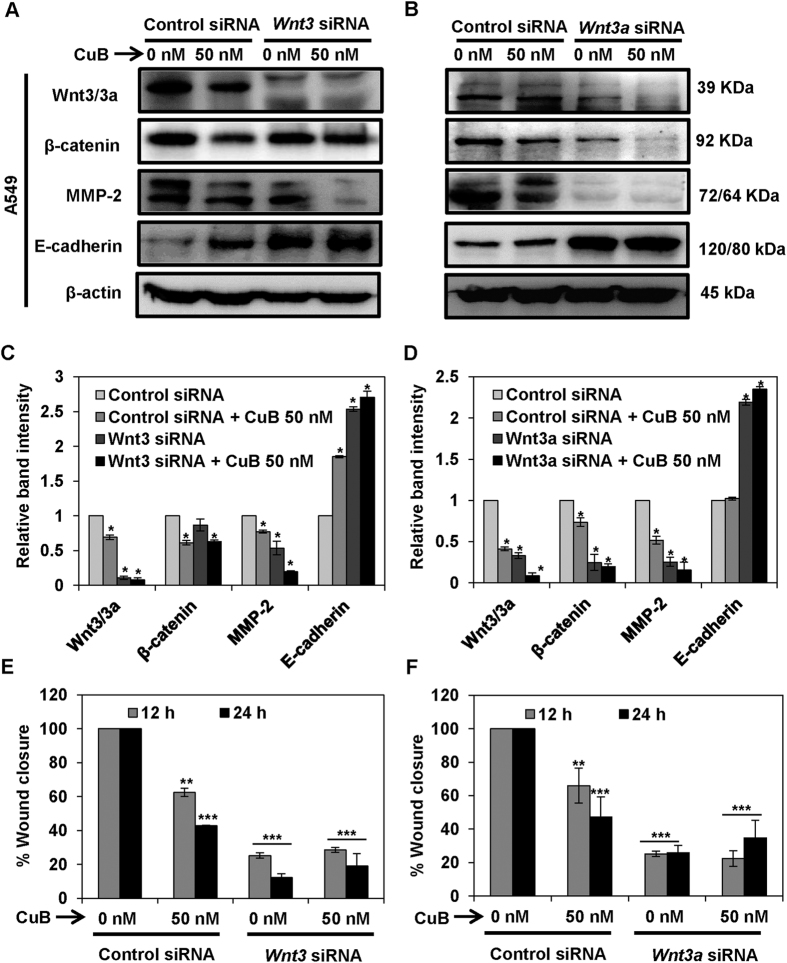
Wnt/β-catenin signaling is involved in the inhibition of metastatic progression of NSCLC A549 cells. A549 (Panels **A**–**D**) were subjected to treatment with 40 nM of *Wnt3* or *Wnt3a* siRNA, respectively. Control- and *Wnt3/3a*-siRNA transfected cells were treated with the indicated concentrations of CuB for 24 h. Cell lysates were prepared and protein expressions were analyzed by western blot. Blots are representative of three independent experiments. Graphs represent the relative band intensities normalized with β-actin. A549 (Panels **E**,**F**) cells transfected with *Wnt3/3a*-siRNA were treated with indicated concentrations of CuB for 24 h. Cellular migration was determined and plotted against percent wound closure in control group. Results were obtained from three independent experiments, mean ± SEM, Statistical significance, *p < 0.05, **p < 0.01 and ***p < 0.001 against control siRNA group.

**Figure 7:**
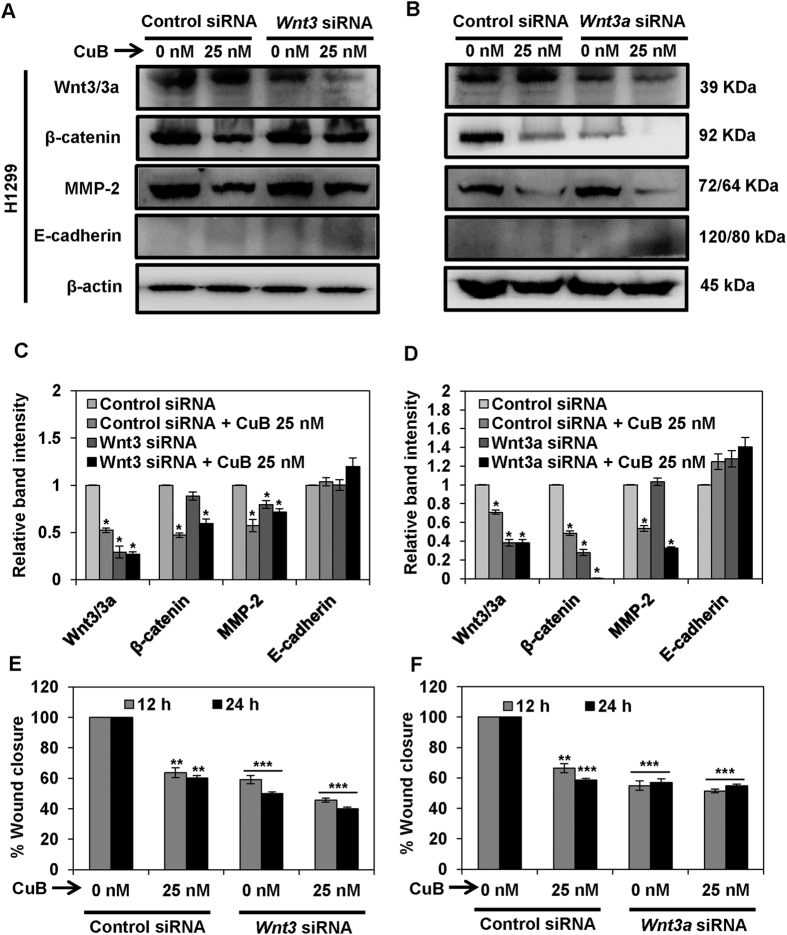
Wnt/β-catenin signaling is involved in the inhibition of metastatic progression of NSCLC H1299 cells. H1299 cells (Panels **A**–**D**) were subjected to treatment with 40 nM of *Wnt3* or *Wnt3a* siRNA, respectively. Control- and *Wnt3/3a*-siRNA transfected cells were treated with the indicated concentrations of CuB for 24 h. Cell lysates were prepared and protein expressions were analyzed by western blot. Blots are representative of three independent experiments. Graphs represent the relative band intensities normalized with β-actin. H1299 (Panels **E**,**F**) cells transfected with *Wnt3/3a*-siRNA were treated with indicated concentrations of CuB for 24 h. Cellular migration was determined and plotted against percent wound closure in control group. Results were obtained from three independent experiments, mean ± SEM, Statistical significance, *p < 0.05, **p < 0.01 and ***p < 0.001 against control siRNA group.

To validate the involvement of CuB-mediated inhibition of Wnt/β-catenin signaling axis in the molecular mechanism of action of CuB, we silenced the expression of *Wnt3* and *Wnt3a* through siRNA-knockdown. The Wnt3 and Wnt3a ligands have been previously known to differentially stimulate proliferation and neurogenesis by canonical Wnt/β-catenin signaling^26^,^27^. As shown in Fig. 6A–D, in the A549 cell transfected with control siRNA, marginal downregulation of markers of Wnt/β-catenin pathway, β-catenin and MMP-2 was observed, while in both *Wnt3* and *Wnt3a* siRNA-transfected A549 cells, these proteins were prominently downregulated. In the *Wnt3*-silenced A549 and H1299 cells, the expression of β-catenin and E-cadherin are comparable to their protein expressions in control-siRNA treated cells. However, in the *Wnt3a*-silenced NSCLC cells, the inhibition of β-catenin expression and upregulation of E-cadherin expression is higher than the control siRNA-transfected cells. Our results suggest that the contribution of Wnt3a in the inhibition of Wnt/β-catenin signaling is higher as compared to Wnt3 in case of NSCLC. Although silencing of *Wnt3/3a* has been shown to alter the expression of MMP-2 and E-cadherin, CuB further pronounced the effects on these proteins. The reason behind this effect might be that CuB has been known as a potent STAT-3 inhibitor and STAT-3 regulates the expressions of both E-cadherin and MMP-2^28^,^29^,^30^,^31^,^32^. Therefore, the effects of CuB are higher in comparison to the effects of Wnt silencing, which functions exclusively through inhibition of Wnt/β-catenin signaling. We next analyzed the effects of *Wnt3/3a* silencing on the migratory characteristics of A549 cells and we found that these effects were comparable to that of changes in the protein expressions. *Wnt3/3a* silencing induced a significant decrease in the cellular migration similar to the CuB-treated control-siRNA group. The anti-migratory effects in Wnt3/3a-silenced, CuB-treated cells were comparable to the effects in either of the treatment groups (p < 0.01; Fig. 6E,F; Supplementary Fig. S3). In H1299 cells, in addition to the inhibition of protein expression induced through *Wnt3* or *Wnt3a* silencing, CuB further downregulated the expression of proteins associated with Wnt/β-catenin signaling. Expression of E-cadherin with CuB treatment in control siRNA-transfected H1299 cells or *Wnt3/3a* siRNA-transfected cells was not observed, which might be due to the lower concentrations of CuB (25 nM) used in this experiment (Fig. 7A–D). We further analyzed the effects of *Wnt3/3a* silencing on the migratory characteristics of H1299 cells. We found that the silencing of *Wnt3*/*3a* induced a significant inhibition of migration in H1299 cells and these effects were almost similar to the CuB-treated, *Wnt3/3a* siRNA-transfected cells, where no further inhibition of migration was observed (p < 0.01; Fig. 7E,F, Supplementary Fig. S4). Collectively, these results validate that CuB-mediated suppression of lung cancer metastasis is mediated, at least in part, through the down regulation of Wnt/β-catenin signaling axis in NSCLC.

To further validate the effect of CuB on β-catenin activation, we performed co-immunoprecipitation analyses. The interaction of nuclear β-catenin with TCF/LEF family of transcription factors is crucial to achieve the activation of downstream molecular signaling. As shown in Supplementary Fig. S5, we found that CuB treatment reduces the interaction of β-catenin with TCF-1 in both A549 and H1299 cells after 24 h. These results validate the observation that CuB-mediated inhibition of nuclear translocation of β-catenin restricts its interaction with transcription factor TCF-1, thereby leading to downregulation of expression of downstream molecular markers.

### CuB downregulates Wnt/β-catenin signaling axis in NNK-induced lung tumorigenesis

**Figure 8:**
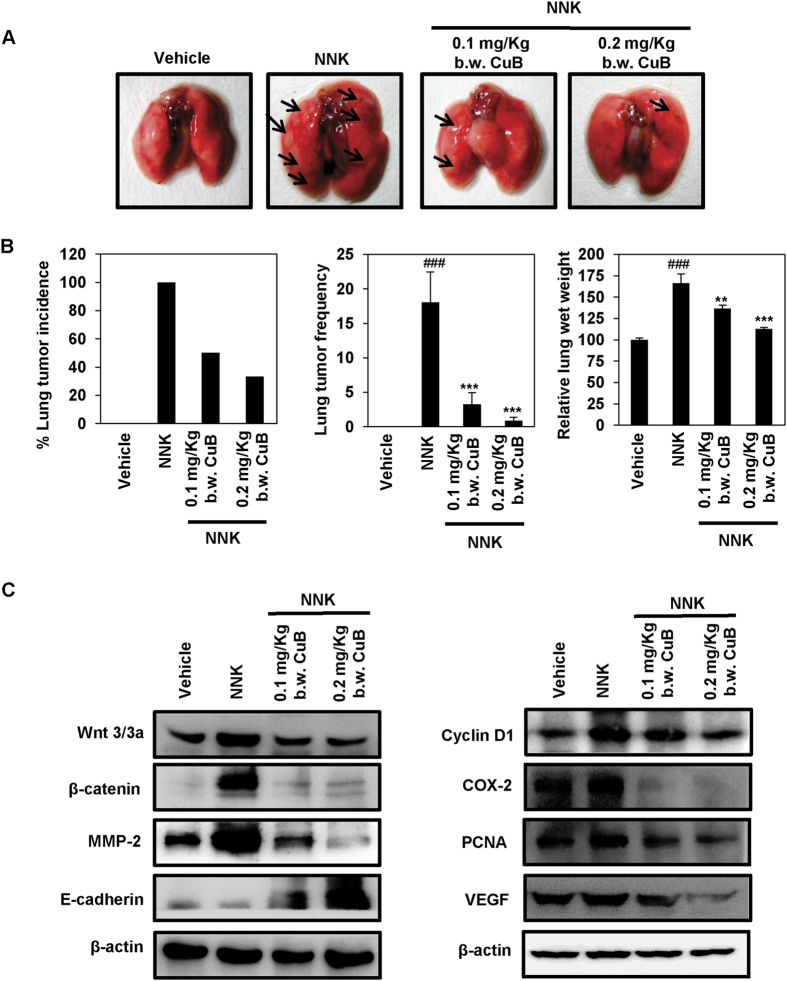
CuB inhibits NNK-induced lung tumorigenesis and Wnt/β-catenin/MMP-2 signaling. (**A**) Representative images of vehicle-treated, NNK-induced CuB-untreated as well as 0.1 mg/Kg and 0.2 mg/Kg b.w. CuB-treated mice lungs. (**B**) Effects of CuB on lung tumor incidence, lung tumor frequency and relative lung wet weights in NNK-treated mice. mean ± SEM. ^**###**^p < 0.001 compared to vehicle-treated lungs, **p < 0.01 and ***p < 0.001 compared to NNK-induced lungs. (**C**) Tissue lysates from each animal group were used to analyze the expression of different Wnt/β-catenin signaling markers. β-actin was used as equal loading control. Blots are representative of three independent experiments.

NNK-induced A/J mouse model has been widely used to study the chemotherapeutic potential of various compounds against lung carcinogenesis^6^,^33^. To validate our *in vitro* results, we assessed the effect of CuB treatment against NNK-induced lung carcinogenesis and the expression of metastasis and stemness regulatory markers. As shown in Fig. 8A,B, administration of NNK significantly induced lung tumor incidence, lung tumor frequency and average wet weight of lungs compared to the vehicle-administrated mice. Treatment of NNK-induced mice with 0.1 and 0.2 mg/Kg b.w. CuB led to the significant reduction in the tumor frequency up to 3.2 ± 1.73 and 0.8 ± 0.5 per animal accounting for ~82% reduction and ~95% reduction in the tumor frequency (p < 0.001). Similarly, the relative wet weights of the lungs of NNK-induced animal groups were found to be increased by more than 65%. NNK-induced CuB-treated animals showed reduction in the lung wet weight (Fig. 8A,B). We did not observe any metastatic lesions in other parts of the body including bone in these mice. We further assessed the effect of CuB treatment on the expression patterns of Wnt/β-catenin signaling mediators. CuB treatment also significantly reduced the β-catenin expression and nuclear localization in NNK-induced A/J mice lungs (p < 0.001; Supplementary Fig. S2B and S2D). As shown in Fig. 8C, the expression of Wnt3/3a, β-catenin and MMP-2 were higher in the NNK-induced group. CuB treatment dose-dependently inhibited the expression of these proteins. The E-cadherin expression was considerably upregulated with the CuB treatment, which further validates our *in vitro* results. Similarly, the expressions of Cyclin D1, COX-2, PCNA and VEGF were also downregulated in the NNK-induced mice lungs following the treatment with 0.1 mg/Kg and 0.2 mg/Kg b.w. of CuB. Collectively, our *in vivo* results further support that the anti-metastatic and anti-angiogenic effects of CuB, as observed *in vitro*, are mediated through the inhibition of Wnt/β-catenin signaling axis in NSCLC.

## Discussion

Malignant progression is characterized by the alterations in normal cellular behavior leading to deregulated proliferation, migration, invasion and angiogenic capabilities, thereby facilitating cancer metastasis. Since metastases pose a potential threat for the recurrence and mortality in the lung cancer patients, development of newer potential therapeutics for metastatic lung cancer is highly required. Recently, we have shown CuB-mediated anti-lung cancer effects both *in vitro* as well as *in vivo*. CuB was found to alter the promoter methylation and histone modifications in the key tumor-related genes at nanomolar concentrations leading to the inhibition of cellular proliferation and induction of apoptosis in NSCLC cells as well as in NNK-induced lung cancer^6^. In our present study, we have demonstrated that in addition to its anti-proliferative activity, CuB also possesses potential anti-metastatic and anti-angiogenic activities in NSCLC.

We found that CuB inhibited the migration and invasion of moderately metastatic as well as highly metastatic NSCLC cells, at sub-toxic concentrations of nanomolar range through inhibition of Wnt/β-catenin signaling. The stem cell-like populations present in the cancer cell pools are characterized by the formation of *in vitro* tumorspheres and colonies. Studies have reported that the inhibition of stemness of cancer cells reduces the chemo-resistance and aggressiveness of cancer^34^,^35^,^36^. CuB treatment potentially inhibited the stemness of the human NSCLC cells at very low concentrations. β-catenin is a central regulator of development, stem cell characteristics as well as cancer metastasis^10^. In the epithelial cells, β-catenin regulates the cell growth and intracellular adhesion by functioning as a component of cadherin protein complexes. Due to its central role in the regulation of metastatic, stem-cell specific and angiogenesis-related pathways, we analyzed the effect of CuB treatment on the expression as well as nuclear localization of β-catenin in NSCLC cells. Interestingly, CuB inhibited both the expression and the nuclear localization of β-catenin in NSCLC.

Canonical Wnt signaling includes the activation of β-catenin downstream pathway by different Wnt ligands. Wnt3 and Wnt3a are important Wnt ligands which have been shown to be upregulated in case of NSCLC^9^,^10^,^11^,^26^,^27^. These ligands bind with FZD-7 receptor and activate the downstream β-catenin signaling. We found that the expressions of both Wnt3 and Wnt3a were inhibited by the CuB treatment. We observed that the expression of FZD-7 receptor was significantly higher in the CuB-treated NSCLC cells. FZD-7 is a seven-pass transmembrane receptor, which belongs to G-protein coupled cell surface receptor (GPCR) family. Previously, it has been demonstrated that the members of GPCR family have the potential to remain active even in the absence of their ligand. Receptor density has been positively correlated to spontaneous activity and various classes of GPCRs have been shown to display constitutive activity upon overexpression^37^. Therefore, the CuB-mediated FZD-7 overexpression could probably be a defence mechanism of the NSCLC cell lines to overcome the loss of Wnt3/3a ligands. Cytoplasmic β-catenin remains sequestrated in the multi-enzyme degradation complex, of which GSK-3 is an important constituent. GSK-3α/β-mediated β-catenin phosphorylation leads to its ubiquitination followed by proteasomal degradation^14^,^37^,^38^,^39^. We found that CuB inhibits the inactivating phosphorylations of both GSK-3α and GSK-3β in NSCLC cells. In addition, the expression of GSK-3α and GSK-3β was found to be upregulated in both A549 and H1299 cells following CuB exposure. Previous studies have also shown that the upregulation of GSK-3 might also lead to activation of apoptotic signaling in cancer^40^. The significantly lower phosphorylation as well as significantly higher GSK-3α/β expression in these two NSCLC cells might be the reason for the loss of β-catenin expression in these cells. Wnt3/3a binding to FZD-7 receptors leads to the inhibition of GSK-3β-mediated phosphorylation of β-catenin by displacing GSK-3β from the β-catenin degradation complex. These β-catenin molecules get accumulated in the cytoplasm and then are translocated into the nucleus, where they stimulate the expression of genes related to cellular proliferation (Cyclin D1), stem cell characteristics (MYC), metastasis (MMPs and E-cadherin) as well as angiogenesis (Survivin and VEGF)^10^. CuB-mediated inhibition of β-catenin signaling further reduces the expression of MMP-2, Cyclin D1, MYC, Survivin and VEGF; and induces E-cadherin expression, thereby hindering the metastatic and stemness behavior of human NSCLC. Further, siRNA transfection of *Wnt3/3a* validated the involvement of canonical Wnt/β-catenin signaling in the CuB-mediated anti-metastatic effects. Interaction of β-catenin with TCF-1 is an essential step in Wnt/β-catenin signaling, which leads to upregulation of downstream genes. Co-immunoprecipitation analysis of β-catenin/TCF-1 complex also validated CuB-mediated downregulation of Wnt/β-catenin signaling. Thus, our findings are in accordance with previous studies, where inhibition of β-catenin expression and nuclear localization has been shown to downregulate MMPs, further inhibiting tumor cell migration and tumor angiogenesis^41^,^42^.

E-Cadherin/β-catenin complex plays an important role in maintaining the epithelial phenotype and disruption of this complex not only affects the cell-cell adhesion, but also the Wnt/β-catenin signaling^43^. The increase in the nuclear translocation of β-catenin has been correlated with decreased E-cadherin expression^43^. CuB induced the expression of E-cadherin in the NSCLC cells, thereby inhibiting the migratory characteristics of these cells. Survivin, a downstream target of β-catenin, further regulates the expression of VEGF, thereby affecting tumor angiogenesis^23^. CuB was found to inhibit the expressions of both Survivin and VEGF, which correlates well with the anti-tumor and anti-angiogenic characteristics of this compound. We observed that CuB was more potent in the H1299 cells with mesenchymal morphology as compared to the A549 cells, which display epithelial morphology. TGF-β1 has previously been reported to induce EMT in A549 cells^24^,^25^. β-catenin plays an important role in the TGF-β1-dependent induction of EMT^44^. In the absence of TGF-β1, β-catenin is rapidly degraded following contact disassembly from the E-cadherin/β-catenin complex. However, in presence of TGF-β1, this dissociated β-catenin is stabilized in the cytoplasm, making it available for nuclear import^44^,^45^. Therefore, we utilized the TGF-β1-induced experimental EMT model to analyze if the morphology of cancer cells affects the potentiality of CuB treatment. Interestingly, CuB was found to be more effective in inhibiting the migratory abilities of TGF-β1-induced A549 cells with mesenchymal characteristics. We found higher inhibition of β-catenin downstream signaling in TGF-β1-induced CuB-treated A549 cells.

Tumor angiogenesis is another important event during metastatic progression, in which the tumor cells induce the formation of new blood vessels in and around the primary tumor. These blood vessels serve a dual role in facilitating tumor progression; that of nutrient and oxygen supply and as passages for the metastatic tumor cells to reach to more favoured secondary metastatic sites^46^. CuB was found to inhibit the migratory and invasive characteristics of endothelial HUVECs. In addition, CuB also disrupted the formation of tubular structures in HUVECs *in vitro* as well as reduced the pre-existing vasculature *ex vivo*. The *in vitro* and *ex vivo* anti-angiogenic effects of CuB were validated *in vivo*, where CuB inhibited the microvessel formation in the matrigel plug injected into the mammary fat pad of Balb/C mice more potentially than imatinib, a standard anti-angiogenic drug. These effects were well correlated with the CuB-mediated inhibition of angiogenesis mediators MMP-2, Survivin and VEGF in NSCLC cells as well as MMP-2 and VEGF in the NNK-induced lung tumors.

**Figure 9:**
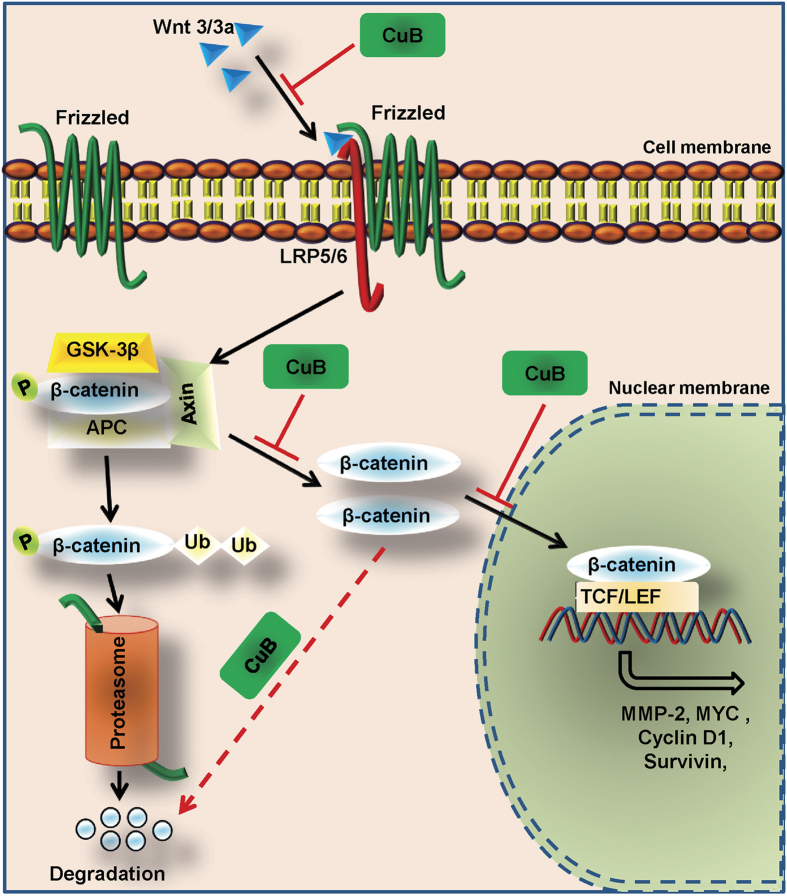
Schematic representation of the mechanism of action of CuB against the metastatic abilities of NSCLC. CuB inhibits the expression of canonical Wnt ligands, which leads to the degradation or inactivation of β-catenin. This results in the reduction of nuclear translocation of β-catenin leading to inhibition of metastasis and angiogenesis.

The key ingredient of tobacco smoke, NNK systemically induces lung tumors in mice, rats and hamsters^33^. NNK-induced mice lung tumors are physiologically very similar to those induced in human through tobacco smoking^46^,^47^. This model has successfully been used to study the course of lung tumorigenesis as well as to analyze the capability of different therapeutic interventions in inhibiting the disease incidence and progression^48^,^49^. NSCLC tumors have been previously reported to have high expression as well as higher nuclear localization of β-catenin^11^. Similarly, NNK has also been reported to induce the β-catenin expression and nuclear localization in the lung tumors of A/J mice, which has been correlated with higher metastatic tendencies^50^,^51^. Therefore, we studied the effect of CuB treatment on the activation of Wnt/β-catenin signaling in NNK-induced lung cancer mice model. We found that, in addition to inhibiting the lung tumor incidence and tumor frequency in NNK-induced mice lungs, CuB also inhibits the Wnt/β-catenin signaling *in vivo*. This reduction in primary tumor frequency and downregulation of canonical Wnt/β-catenin signaling inhibits the metastatic abilities of NNK-induced lung tumors *in vivo*. The proposed molecular mechanism of CuB-mediated inhibition of metastatic abilities of NSCLC has been depicted in Fig. 9.

Collectively our findings conclude that, in addition to the previously reported potential anti-proliferative and pro-apoptotic activities, CuB also inhibits NSCLC metastasis and angiogenesis at sub-IC_50_ doses. Further, CuB decreases stemness in NSCLC at sub-IC_50_ concentrations. These potential anti-cancer effects of CuB are mediated, at least in part, through the downregulation of canonical Wnt/β-catenin signaling axis. However, more mechanism-based studies are required using *in vivo* metastatic models with CuB to further validate its potential against NSCLC.

## Methods

### Cell Culture

Human NSCLC A549, H1299 and H23 cells were obtained from American Type Culture Collection (ATCC, Manassas, VA). Cells were cultured and maintained in RPMI-1640 medium (Sigma-Aldrich, St. Louis, MO) supplemented with 10% FBS (Sigma Aldrich) and 1% penicillin-streptomycin solution (Sigma-Aldrich). HUVECs were procured from Life Technologies and maintained in M-200 medium (Invitrogen, Carlsbad, CA) supplemented with LSGS (Invitrogen) and 1% penicillin-streptomycin solution (Sigma-Aldrich). All the cells were grown in a humidified atmosphere at 37 °C with 5% CO_2_. CuB was isolated from *Luffa graviolense* Roxb. and dissolved in ethanol at a stock concentration of 10 mM as described previously^6^.

### Wound healing assay

The migratory capacities of lung adenocarcinoma A549, H1299 and H23 cells were assessed by *in vitro* wound healing assay as described previously^52^. Briefly, 2 × 10^5^ cells were seeded in 6-well plates and grown to a confluency of 80–90%. A wound was created in the middle of the well and cells were treated with different concentrations of CuB for 12, 24 and 48 h. Wound closure photographs were captured using Nikon’s Eclipse TS100 microscope equipped with a digital camera. The wound area was measured by using ImageJ 1.46r software. The results were expressed as percent wound closure compared with control.

### Matrigel Invasion assay

The invasive capabilities of NSCLC cells were assessed by invasion assay using Boyden chambers (BD Biosciences) as described previously^52^. Approximately, 2 × 10^4^ cells were seeded in the upper chamber in 500 μL of medium supplemented with 1% FBS containing 0–100 nM concentrations of CuB. In the lower well, 750 μL medium supplemented with 10% FBS was added as a chemoattractant. Cells were incubated at 37 °C in the CO_2_ incubator for 48 h. After incubation, cells on the lower surface of the membrane were fixed and stained with 0.1% crystal violet (Sigma–Aldrich). The membranes were photographed and the numbers of invaded cells were counted in at least 10 different microscopic fields. Data were presented as percent invasion compared to respective controls.

### Tumorsphere assay

A tumorsphere is defined as a solid spherical mass developed from the proliferation of a single cancer stem cell (CSC) or a progenitor cell. Tumorsphere assay is used to estimate the percentage of cancer stem/progenitor cells present in a population of tumor cells^53^. Tumorsphere assay was performed as described previously^54^. Approximately, 200 cells/well in 10 replicates were seeded in ultra-low attachment 96-well plates in DMEM/F12-medium supplemented with 1× B27 supplement (Invitrogen), 20 ng/mL epidermal growth factor, 10 ng/mL basal fibroblast growth factor, 5 μg/mL insulin, 0.4% bovine serum albumin (Sigma Aldrich). The upper and lower edges of the 96-well plate were sealed and cells were incubated undisturbed in a CO_2_ incubator for one week. Thereafter, tumorsphere frequency was counted under a phase-contrast microscope at 400× magnification. Results were represented as the average frequency of tumorspheres per well in the vehicle as well as treatment groups.

### Clonogenic assay

*In vitro* colony forming potentials of NSCLC cells were assessed by anchorage-dependent clonogenic assay, as described previously^52^,^55^. Approximately 500 cells were seeded into 6-well plates in triplicates. After 24 h, cells were treated with different CuB concentrations and were allowed to incubate at 37 °C in the CO_2_ incubator for 15 days. After incubation, the colonies were fixed and stained with 0.1% crystal violet (Sigma-Aldrich). The colonies with ≥50 cells were counted and expressed as percent control.

### Confocal immunofluorescence imaging

The immunofluorescence experiments were performed as described previously^56^,^57^. Approximately, 2 × 10^4^ cells were treated with 0, 25, 50 and 100 nM of CuB and incubated in CO_2_ incubator for 24 h. Then after, the cells were fixed in a freshly prepared 2% paraformaldehyde and autofluorescence quenching was performed by incubating the cells with ammonium chloride at room temperature. Cells were permeabilized using 0.1% Triton X-100 and non-specific sites were blocked by incubating the cells with 2% BSA for 45 min at room temperature. The cells were then incubated with anti-β-catenin primary antibody (Invitrogen; 1:200) at 4 °C overnight. Following washing, the cells were incubated in FITC-conjugated anti-rabbit secondary antibody (Sigma-Aldrich, 1:160) for 1 h in dark at RT. After washing, the nuclei were counterstained using DAPI (0.5 μg/mL). IgG control was used as a negative control of immunofluorescence staining. The fluorescent images of CuB-treated and -untreated A549 and H1299 cells at 630× magnification were captured using confocal microscope (Carl Zeiss, Germany). The fluorescent images of CuB-treated and -untreated H23 cells as well as NNK-induced lung tissues were captured using fluorescence microscope (Carl Zeiss). The fluorescent intensity of NSCLC cells was represented as raw integrated density and corrected total cell fluorescence (CTCF). CTCF was calculated as; Integrated Density - (Area of selected cell × Mean fluorescence of background readings) and represented as an average value of total number of cells analyzed (indicated in graphs) as described previously^58^. The average fluorescence intensity of NNK-induced tumors was measured as the fluorescence intensity of lung tissue analyzed in at least five different microscopic fields.

### siRNA knockdown of *Wnt3*/*3a*

The siRNA transfection experiments were performed as described previously^59^. Approximately 2 × 10^5^ cells per well were seeded in a 6-well plate and allowed to incubate overnight. Mission esiRNA for *Wnt3* and *Wnt3a* (Sigma-Aldrich) were dissolved at a stock concentration of 10 μM using RNase-free water and 40 nM siRNAs were delivered to the cells using the lipofectamine reagent (Invitrogen) according to the manufacturer’s protocol. RLUC negative control siRNA (Sigma-Aldrich) was used as a negative control to monitor knock-down efficacy and toxicity, if any. Cells were treated with CuB for an additional 24 h. Cells were harvested and checked for *Wnt3-* and *Wnt3a-*mediated β-catenin signaling as well as migration, as described in the previous sections.

### Co-immunoprecipitation

Co-immunoprecipitation experiments were performed in whole cell extracts of CuB-treated A549 and H1299 cells. For immunoprecipitation analysis, 50 μL of Sepharose A beads (Sigma-Aldrich) were incubated with 2.5 μg of β-catenin (Invitrogen: 71-2700) antibody diluted up to 500 μL in wash buffer containing PBS with 2% triton-X-100 overnight at 4 °C. After centrifugation and washing, these antibody-coated beads were incubated with 500 μg of whole cell extracts from each sample for 1 h at 4 °C. After, centrifugation, the supernatant was collected. β-actin was used as an equal loading control. Beads were washed thrice and equal volumes of protein from each sample were mixed with laemmlli buffer, boiled in a water bath and loaded onto 12% SDS-PAGE. Immunoblotting analysis was performed by using TCF-1 (Life Technologies: A13969) antibody. The blots were incubated with specific HRP-conjugated secondary anti-rabbit antibody and bands were visualized using ECL (Millipore) on ImageQuant LAS4010 chemiluminescence detection system (GE Healthcare, Amarsham, UK).

### Tube formation assay

The anti-angiogenic potential of CuB was determined by performing tube formation assay in HUVEC cells. HUVECs were seeded on the matrigel pre-coated 96-well cell culture plate at a density of approximately 2 × 10^4^ cells per well and serum starved for 2 h. VEGF (20 ng/mL) was added to each well as an inducer of angiogenesis. After 2 h, cells were treated with indicated concentration of CuB and after an additional incubation of 6 h, photographs were captured. Tubulogenesis was analyzed using ImageJ software version 1.47 h (http://imagej.nih.gov/ij). Total number of nodes and branches were calculated and data were represented as average number of nodes and branches compared to controls.

### Chick Chorioallantoic Membrane (CAM) assay

To assess the effect of CuB on the pre-existing vasculature, we performed *ex vivo* CAM assay according to the previously described method with slight modifications^60^. Embryonic eggs were incubated in humidified (65–70%) chamber at physiological temperature for at least 3–4 days with their CAM exposed to visualize the angiogenesis. At 8^th^ day, 25 nM CuB and PBS as control were added in sterile rubber rings on the exposed CAM and window-sealed eggs were incubated for an additional 2 days. After incubation, the microscopic examination of CAM was performed.

### Western blot analysis

Whole cell lysates of A549 and H1299 cells at 24 h of CuB treatment were prepared using the RIPA-lysis buffer (Millipore, Billerica, MA). Protein extraction from tumor samples was also performed using RIPA-lysis buffer. Proteins were resolved on 10–12% SDS-polyacrylamide gels and transferred onto PVDF membranes (Millipore). After incubation in blocking buffer (5% skimmed milk or 2% BSA in TBST) for 1 h, the membranes were incubated with primary antibodies specific for Wnt3/3a (Abcam: ab172612), Fzd-7 (Sigma-aldrich: AV41251), phospho-GSK-3α (Cell Signaling Technology: 9337), GSK-3α (Abcam: ab40870), phospho-GSK-3β (Santa Cruz: SC-11757), GSK-3β (Abcam: ab18893), β-catenin (Invitrogen: 71-2700), TCF-1 (Life Technologies: A13969), MMP-2 (Cell Signaling Technology: 4022S), E-cadherin (Santa cruz: SC-8426), COX-2 (Millipore: AB5118), MYC (Millipore: 06-340), Cyclin D1 (Santa cruz: SC-8396), Survivin (Cell Signaling Technology: 2808S), VEGF (Santa cruz: S C-53462), Vimentin (Santa cruz: SC-6260), PCNA (Santa cruz: SC-7907) and β-actin (Cell Signaling Technology: 4970L). The blots were then incubated with specific HRP-conjugated secondary antibodies and bands were visualized using ECL (Millipore) on ImageQuant LAS4010 chemiluminescence detection system (GE Healthcare, Amarsham, UK). The band intensities were quantified using ImageJ 1.46r software.

### Animal experiments

Female, 5–6 weeks-old A/J mice were procured and the study was conducted according to the guidelines as well as protocol approved by the Institutional Animal Ethics Committee (IAEC), CSIR-CDRI, India. Briefly, after one week of acclimatization, mice were randomly divided into four groups (n = 8); three groups were injected intraperitoneally (i.p.) with 100 mg/Kg body weight (b.w.) NNK dissolved in saline (Sigma). On third week onwards, NNK-administered mice were treated with 0.1 or 0.2 mg/Kg b.w. of CuB i.p. twice a week, as described previously^6^, except that in this study, the CuB treatment was continued throughout the study period. At 21^st^ week, mice were sacrificed and lungs were examined for tumor incidence, multiplicity and lung wet weight. Lung tissues from each group were snap-frozen in liquid nitrogen and were also fixed in 10% of neutral buffered formalin for histopathological and immunofluorescence analyses.

### *In vivo* matrigel plug assay

Animals were randomly segregated into four groups (n = 6). Matrigel (400 μL) was injected subcutaneously into the mammary fat pads of female Balb/c mice forming semi-solid plugs. Two groups received intraperitoneal (i.p.) injections of 0.1 mg/kg and 0.2 mg/kg of CuB thrice a week for 7 days. A group of animals was injected with imatinib (50 mg/Kg i.p.) as a positive control. On day 7, after anesthetizing the animals, matrigel plugs were excised and fixed in PBS-buffered 10% formalin and then stored for further processing. Following deparaffinization and dehydration, tissue samples were stained with Harris hematoxylin and eosin stains (Sigma–Aldrich). The sections were photographed using Nikon’s Eclipse TS100 microscope equipped with a digital camera. For each group, sections from at least three mice (n = 3) were examined for H&E staining.

### Statistical analysis

The statistical significance of differences between the values of treated samples and controls were determined by One-way ANOVA with Dunnet’s post-hoc test using GraphPad Prism version 3.00 for Windows software. In each case, p < 0.05 was considered statistically significant.

## Additional Information

**How to cite this article**: Shukla, S. *et al*. Cucurbitacin B inhibits the stemness and metastatic abilities of NSCLC via downregulation of canonical Wnt/ß-catenin signaling axis. *Sci. Rep.* **6**, 21860; doi: 10.1038/srep21860 (2016).

## Supplementary Material

Supplementary Information
