# Supplementary material for: Cucurbitacin B inhibits the stemness and metastatic abilities of NSCLC via downregulation of canonical Wnt/β-catenin signaling axis
Source: Sci Rep. 2016 Feb 24;6:21860. doi: 10.1038/srep21860 (PMC4764833; doi:10.1038/srep21860)
Supplement: Supplementary Information [file srep21860-s1.pdf]

Supplementary Figures of manuscript entitled:

Cucurbitacin B inhibits the stemness and metastatic abilities of NSCLC via downregulation of canonical Wnt/β-catenin signaling axis

Samriddhi Shukla[1], Sonam Sinha[1], Sajid Khan[1], Sudhir Kumar[2], Kavita Singh[3], Kalyan Mitra[3], Rakesh Maurya[2], Syed Musthapa Meeran[1,4]*

[1]Laboratory of Cancer Epigenetics, Division of Endocrinology, CSIR-Central Drug Research Institute, Lucknow, India.

[2]Division of Medicinal and Process Chemistry, CSIR-Central Drug Research Institute, Lucknow, India

[3]Electron Microscopy Unit, SAIF, CSIR-Central Drug Research Institute, Lucknow, India

[4]Academy of Scientific and Innovative Research (AcSIR), New Delhi, India

***Correspondence to:** Syed Musthapa Meeran, Ph.D., Division of Endocrinology, CSIR-Central Drug Research Institute (CSIR-CDRI), Jankipuram Extn., Sector-10, Sitapur Road, Lucknow-226 031, India.

Phone: +91 522 2772450, Ext 4491

Fax: +91 522 2771941

Email: s.musthapa@cdri.res.in

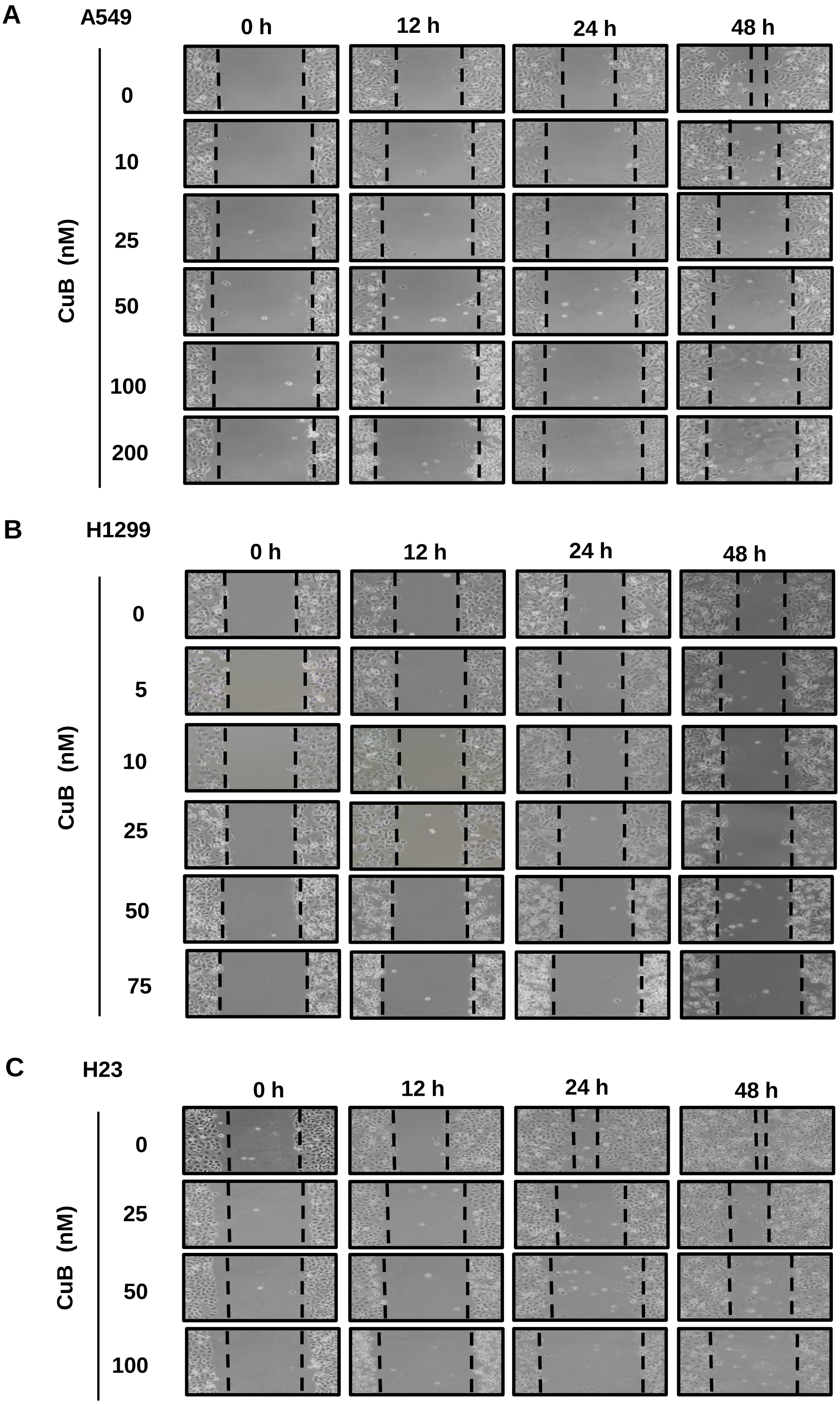

Supplementary Fig. S1

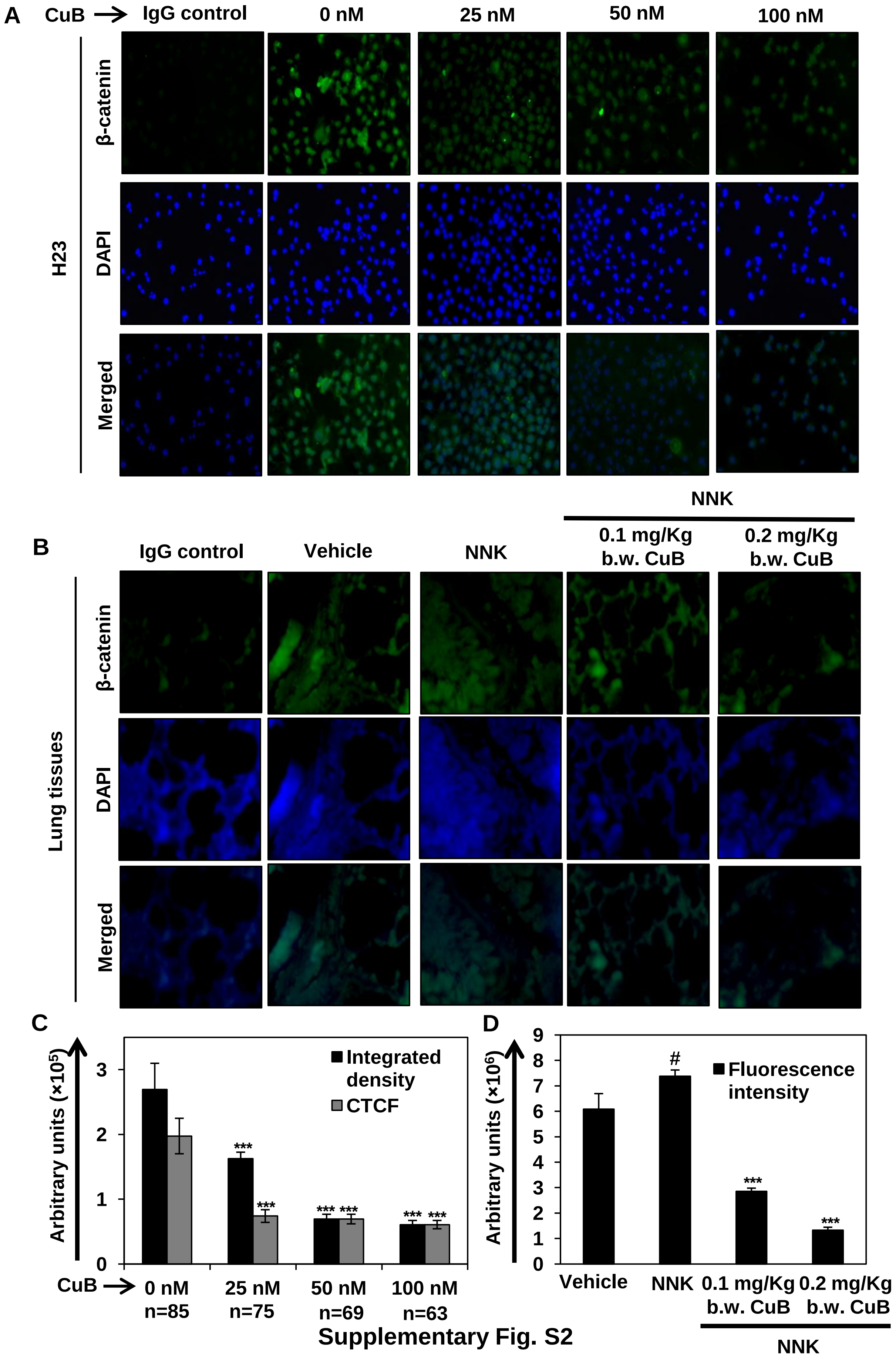

Supplementary Fig. S2

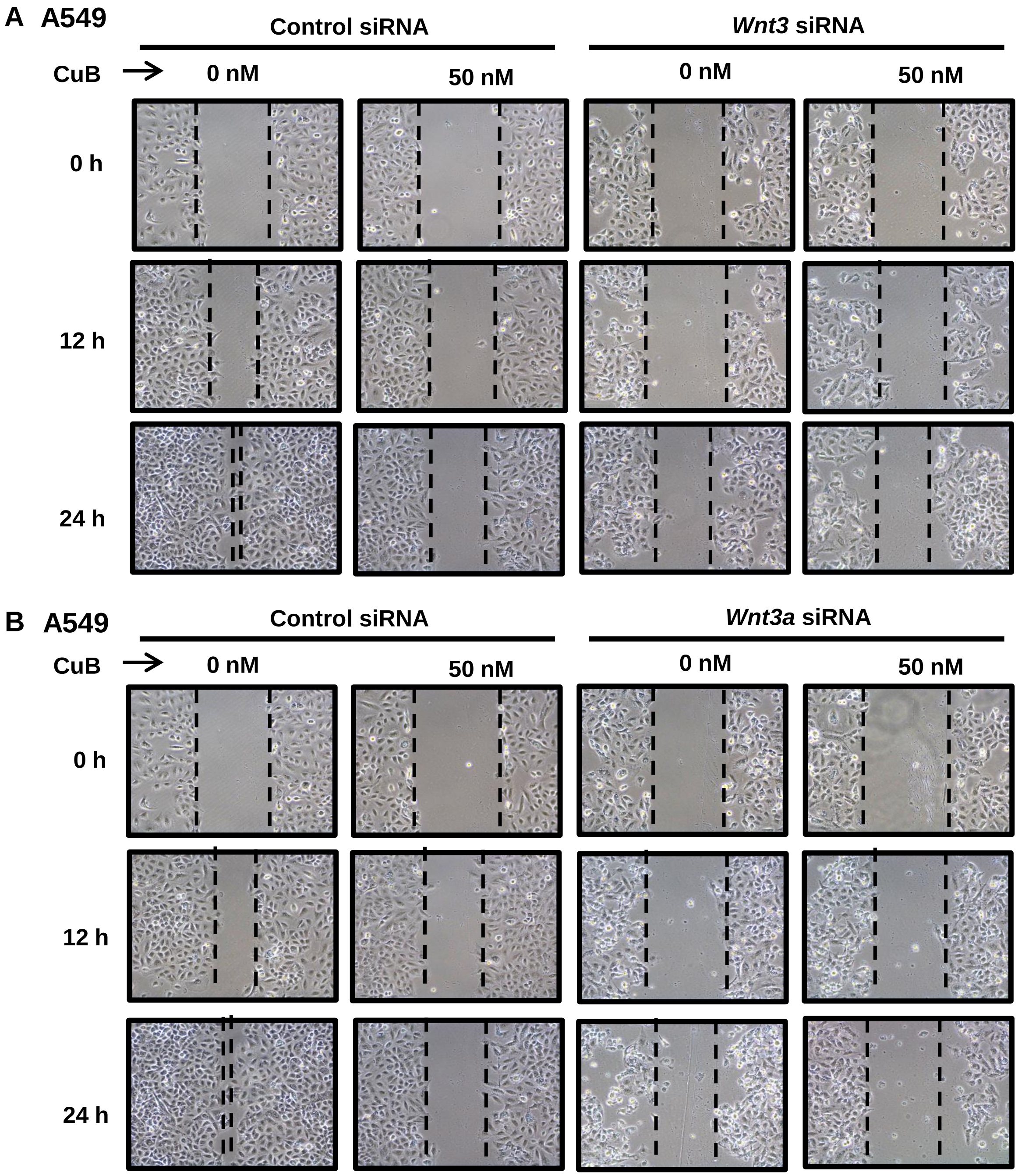

Supplementary Fig. S3

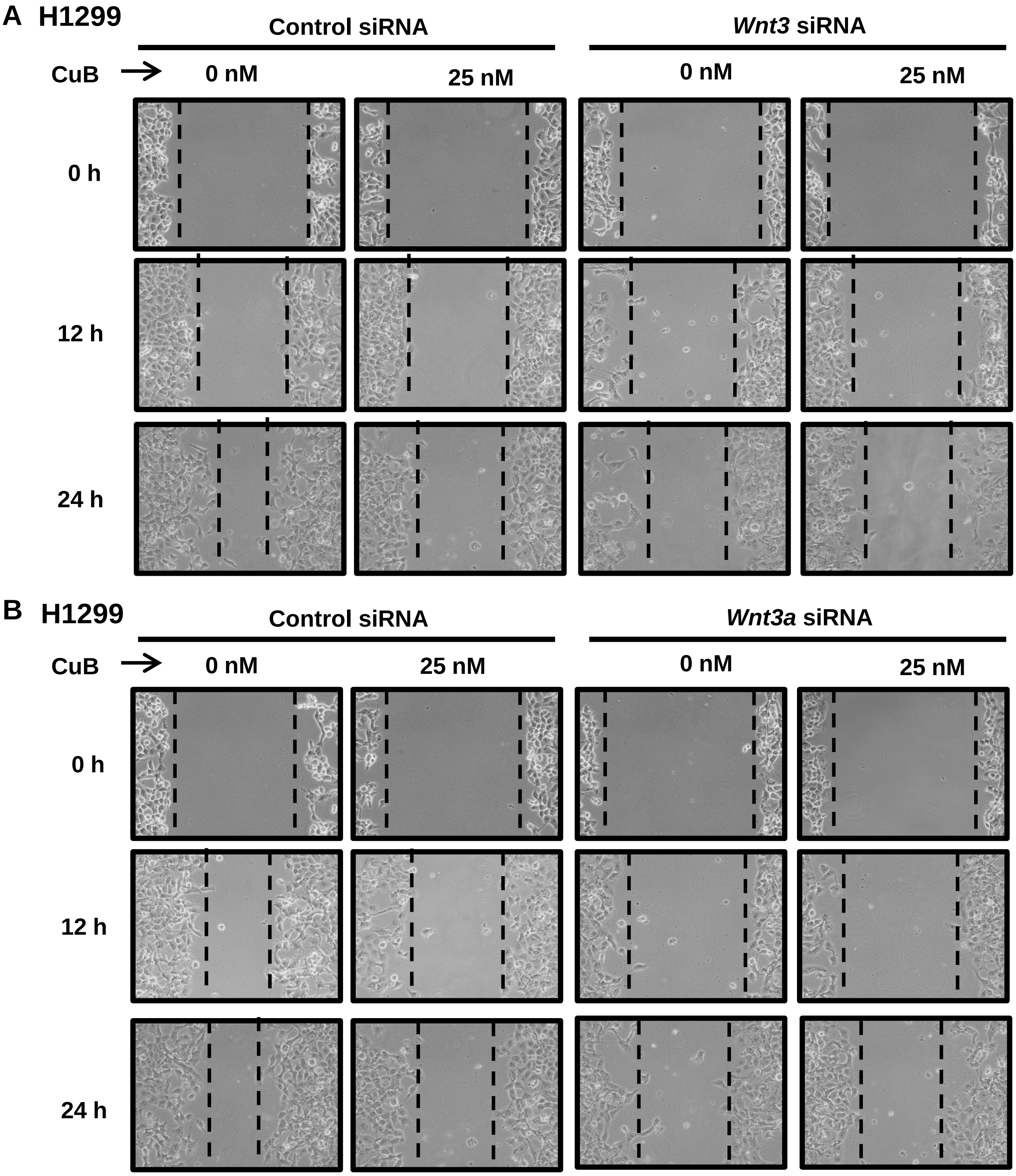

Supplementary Fig. S4

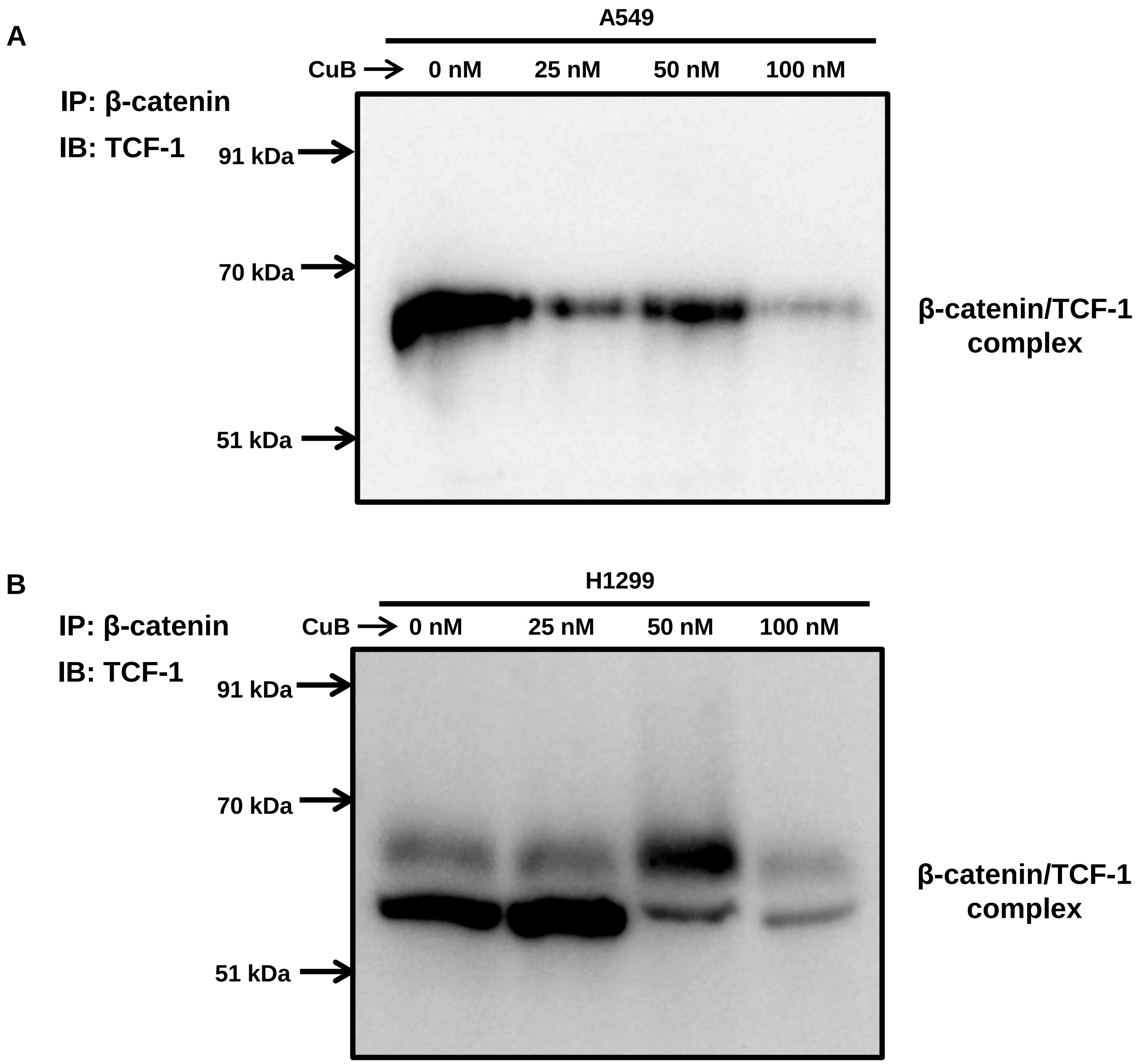

Supplementary Fig. S5

Supplementary Fig. S1. CuB suppresses cellular migration of human NSCLC A549, H1299 and H23 cells at sub-toxic concentrations. The subconfluent A549 (*Panel A*), H1299 (*Panel B*) and H23 (*Panel C*) cells were scratched with a 200 µL pipette tip, and treated with indicated concentrations of CuB for 12, 24 and 48 h. Images are representative of three independent experiments.

Supplementary Fig. S2. CuB inhibits the expression and nuclear translocation of β-catenin in NSCLC H23 cells and in NNK-induced mice lungs. H23 cells (*Panel A*) and NNK-induced CuB- treated lung tissue sections (*Panel B*) were analyzed for eendogenous cytoplasmic and nuclear β-catenin-FITC (green fluorescence) localization through immunofluorescence analysis. Nuclear stain DAPI was used as the counter stain (blue fluorescence). IgG-control was used as a negative control for background staining. Images are representative of three independent experiments. *Panels C* represents the raw integrated density as well as CTCF values in H23 cells. The numbers within the parentheses are indicative of number of cells analyzed. *Panel D* represents the fluorescence intensity of NNK-induced CuB-treated and untreated lungs.

Supplementary Fig. S3. Effect of CuB and *Wnt3/3a* knock down on cellular migration of A549 cells. The subconfluent A549 cells were transfected with *Wnt3/3a* siRNA for 24 h. Then after, cells were scratched with a 200 µL pipette tip, and treated with varying concentrations of CuB for 12 and 24 h. Images of wound closure were captured and wound area were measured after *Wnt3* and *Wnt3a* silencing (*Panel A and Panel B*). Images are representative of three independent experiments.

Supplementary Fig. S4. Effect of CuB and *Wnt3/3a* knock down on cellular migration of H1299 cells. The subconfluent H1299 cells were transfected with *Wnt3/3a* siRNA for 24 h. Images of wound closure in control siRNA- as well as *Wnt3/3a* siRNA-transfected cells were captured and wound area were measured (*Panel A and Panel B*). The images are representative of three independent experiments.

Supplementary Fig. S5. Effect of CuB on the β-catenin/TCF-1 interaction in NSCLC cells. Whole cell extracts of untreated and CuB-treated A549 (*Panel A)* and H1299 (*Panel B)* cells were immunoprecipitated with anti-β-catenin antibody. β-catenin/TCF-1 interaction was studied by immunoblotting with anti-TCF-1 antibody. Blots are representative of three independent experiments.
